# Inside the November 2025 Issue

**DOI:** 10.24908/pocusj.v10i02.20054

**Published:** 2025-11-17

**Authors:** Benjamin T. Galen

**Affiliations:** Professor of Medicine, Albert Einstein College of Medicine and Montefiore, Medical Center, Bronx, NY, USA, Editor-in-Chief, POCUS Journal

**Keywords:** November 2025, Commentary

Dear Readers,

Every issue of POCUS Journal reminds me not only how far our field has come, but also how hard this community works to continue to advance POCUS and patient care.

**Figure:**
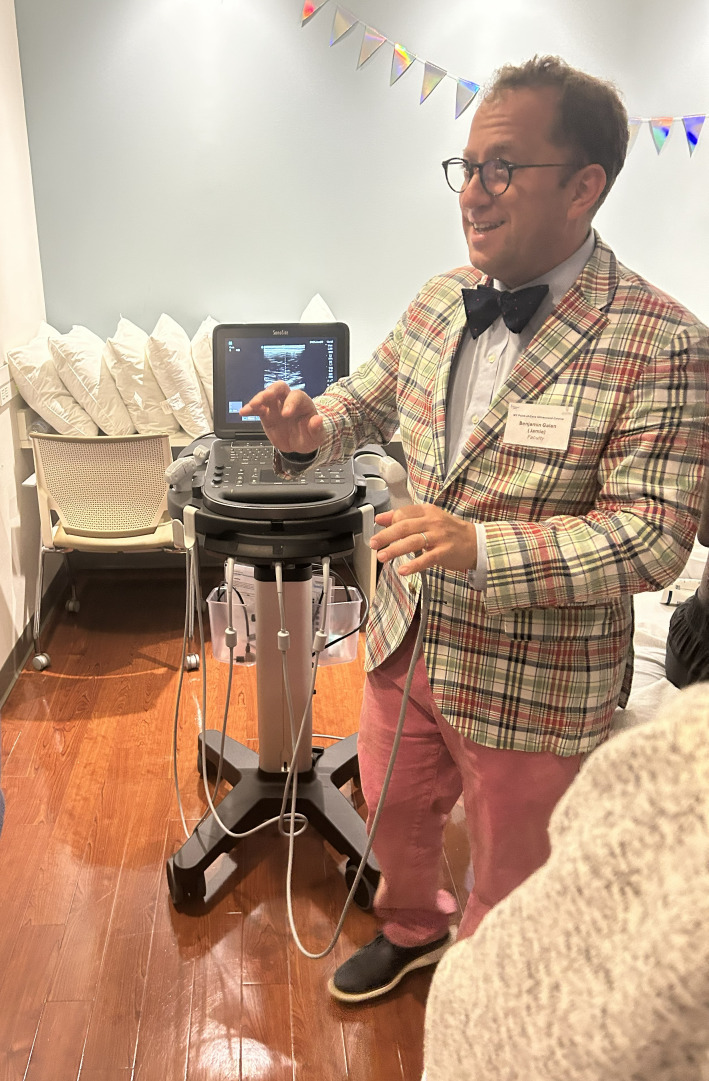
Benjamin T. Galen, Editor in Chief, POCUS Journal

Dr. Sanjay Patel's piece on page 20 POCUS: Presence, Observation, Connection, Understanding, Story" reminds us of the humanity in medicine. His reflection on an encounter early in his training reveals how POCUS images became a turning point for a patient's life. This experience illustrates that POCUS provides a connection for patients that is not present in reading their radiology reports or an after visit summary. POCUS, at its best, does more than help us diagnose patients better and faster; it connects patients and clinicians.

Across the rest of this issue, the breadth of innovation is striking. Practice changing. Can't get a BP by auscultation or automated cuff? Mayo-Malasky et al. (on page 30) show that POCUS with color power Doppler can be used instead. Can't view the IVC in the subxiphoid window? Beltramino et al. (on page 24) provide data that support the transhepatic view of the IVC as a viable alternative.

In addition to late breaking research and novel case reports, this issue boasts an incredibly helpful editorial by Dr. Tanping Wong (of our editorial board), Riya Soni (of the social media team), and Dr. Casey Glass (our deputy editor) titled “A Guide to an Effective Peer Review” on page 7. This guide has so much great advice for peer reviewers that I know it will be an invaluable resource for POCUS Journal and the entire POCUS community for years to come. In fact, their guide and the tables contained therein are not specific to POCUS research and therefore are broadly applicable across many scientific disciplines. I anticipate this guide being shared broadly by journal editors and peer reviewers alike. It is a privilege for POCUS Journal to publish their guide and I am grateful that our reviewers will have resources to help them with the peer review process.

This issue would not be possible without the hard work of our editorial board, our reviewers, and our publisher CINQUILL. A special thanks to Pedro Sartori Manoel, our Managing Editor whose dedication to the editorial process at all steps made this issue possible.

Thank you, as always, for reading, contributing, and continuing to build this global community of POCUS educators, clinicians, and innovators.

Sincerely,

Benjamin T. Galen, MD

Professor of Medicine

Albert Einstein College of Medicine and Montefiore Medical Center, Bronx, NY, USA

Editor-in-Chief,

POCUS Journal

